# Viral culture, genome characterization, and mutation analysis of human influenza A virus

**DOI:** 10.5808/gi.22043

**Published:** 2022-09-30

**Authors:** Rujittika Mungmunpuntipantip, Viroj Wiwanitkti

**Affiliations:** 1 Private Academic Consultant, Bangkok 103300, Thailand; 2 Department of Community Medicine, Dr. D.Y. Patil University, Pune 400706, India

Dear Editor, we found that the publication on “Genome characterization and mutation analysis of human influenza A virus in Thailand” in the journal [1] is very interesting. This study's findings indicated that 90 samples, including 44 H1N1 and 46 H3N2 viruses, were virally positive. Forty-three of these samples were successfully isolated, and 25 of those had their viral genomes entirely amplified. The genetic characterization of influenza viruses that are now in circulation is suggested as the final step in preparing for pandemic and epidemic outbreaks in the future. We all agree that the information is helpful. However, the most accurate laboratory analysis should be used to generate the results. There may be some clinical laboratory significance to the low isolatable rate in this report. The Madin-Darby canine kidney (MDCK) cells were used in the current study's methods for viral culture. According to a prior clinical investigation [2], MDCK-SIAT1 cells outperform conventional MDCK. Utilizing a better cell type for culture may aid in the virus's capacity to be isolated and provide accurate information on the molecular epidemiology of influenza A.
